# Supplementary material for: Skin Wound Healing Process and New Emerging Technologies for Skin Wound Care and Regeneration
Source: Pharmaceutics. 2020 Aug 5;12(8):735. doi: 10.3390/pharmaceutics12080735 (PMC7463929; doi:10.3390/pharmaceutics12080735)
Supplement: Supplementary file 1 [file pharmaceutics-12-00735-s001.pdf]

pharmaceutics

Supplementary Materials: Skin Wound Healing Process and New Emerging Technologies for Skin Wound Care and Regeneration

Erika Maria Tottoli [1], Rossella Dorati [1,*], Ida Genta [1], Enrica Chiesa [1], Silvia Pisani [2] and Bice Conti [1]

[1] Department of Drug Sciences, University of Pavia, V.le Taramelli 12, 27100 Pavia, Italy; erikamaria.tottoli01@universitadipavia.it (E.M.T.); ida.genta@unipv.it (I.G.); enrica.chiesa@unipv.it (E.C.); bice.conti@unipv.it (B.C.);

[2] Immunology and Transplantation Laboratory, Pediatric Hematology Oncology Unit, Department of Maternal and Children's Health, Fondazione IRCCS Policlinico S. Matteo, 27100, Pavia, Italy silvia.pisani01@universitadipavia.it;

* Correspondence: rossella.dorati@unipv.it; Tel.: +390382987393

Table S1. Comparative summary of skin wound care and regeneration emergent technologies (technology, application advantages and disadvantages and examples of commercial products).

Technology	Application	Advantages	Disadvantages	Refs.	Examples of commercial products
**Advanced dressing**	Wound care serious injuries with complex healing processes	Active role in wounds treatment stimulation of healing. Wound healing process Synergistic enhance by growth factors addition	Not suitable for broad spectrum applications. Growth factors low absorption capacity	[29, 47-49, 51, 54-56]	Cutisorb™, Iodosorb, Actisorb Silver 220, Acticoat
**Skin grafts**	Skin regeneration, severe surface deficits	Restoration of integumentary continuity	Invasive procedures that can expose the patient to serious complications	[50]	–
**Cellular skin substitutes Fibroblasts, keratinocytes**	Skin regeneration Deep, non-healing wounds	Actively promote skin regeneration due to their structure and composition Basis for revascularization	Limited donor sites, high risk of secondary morbidity. Cannot be applied to all patients with chronic injuries.	[58 - 61]	Epicel Dermagraft® Apligraf ®
**Cellular skin substitutes Epidermal stem cells and progenitors.**	Skin regeneration Large size, deep, non-healing wounds.	Autologous cell sources for chronic wound healing Safety and easy isolation from tissues Accelerate wound healing, improve the quality of healing, and angiogenesis	Expensive	[51, 63 - 67]	Rigenera system (once upon a time micrografts application)
**Cellular skin substitutes Gene Therapy**	Skin regeneration, Epidermolysis bullosa	Transgenic epidermal Grafts	Doubts on safety and effectiveness.	[68 - 70]	–
**Cellular skin substitutes Induced pluripotent stem cells**	Skin regeneration.	Avoid complication of immune system	Doubts on safety.	[60, 72 - 77]	–

**Cell free scaffolds Decellularization techniques**	Skin regeneration.	Dermal replacement with superior biocompatibility and less immunogenicity	Decellularization protocol negatively impact on matrix structure and orientation	[60, 103, 104]	–
**Cell free scaffolds Electrospinning**	Wound care and skin regeneration.	Nanofibrous polymer membrane with variable pore size, high surface area and oxygen permeability. Combination with drugs (i.e. antibiotic) and antimicrobial loaded nanoparticles.	Not all biomaterials can be electrospun	[105 - 110]	–
**Cellular scaffolds. 3D-bioprinting**	Skin regeneration.	Tissues formed by layers with different cell density able to simulate the multi-tissue structure complexity. Flexibility to control geometry at micro/ nano-cellular level. Modulates cell-cell interaction in specific 3D environment.	Thickness of tissue is not suitable for clinically application.	[111 - 116]	–
